# Supplementary material for: Tropical peatland carbon storage linked to global latitudinal trends in peat recalcitrance
Source: Nat Commun. 2018 Sep 7;9:3640. doi: 10.1038/s41467-018-06050-2 (PMC6128871; doi:10.1038/s41467-018-06050-2)
Supplement: Supplementary file 1 — Supplementary Information [file 41467_2018_6050_MOESM1_ESM.pdf]

Supplementary Information

Tropical peatland carbon storage linked to global latitudinal trends in peat recalcitrance

Hodgkins et al.

Supplementary Figures

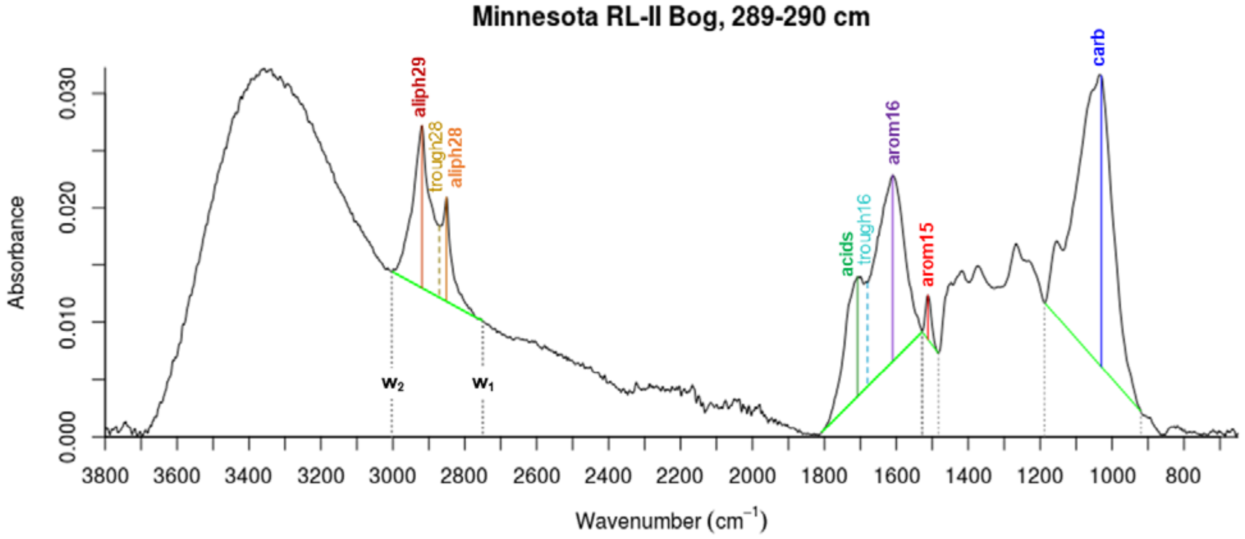

Supplementary Fig. 1 Baseline corrections for Fourier transform infrared spectroscopy peaks. These baseline corrections are illustrated with an example peat sample from the Minnesota RL-II Bog site. Baselines are represented as solid green diagonal lines, baseline-corrected peak heights as solid color-coded vertical lines, and baseline endpoints (labeled for the aliphatic region between 2750–3000 cm^{-1}) as dotted gray vertical lines. Absorbance values are normalized to the integrated area of the spectrum.

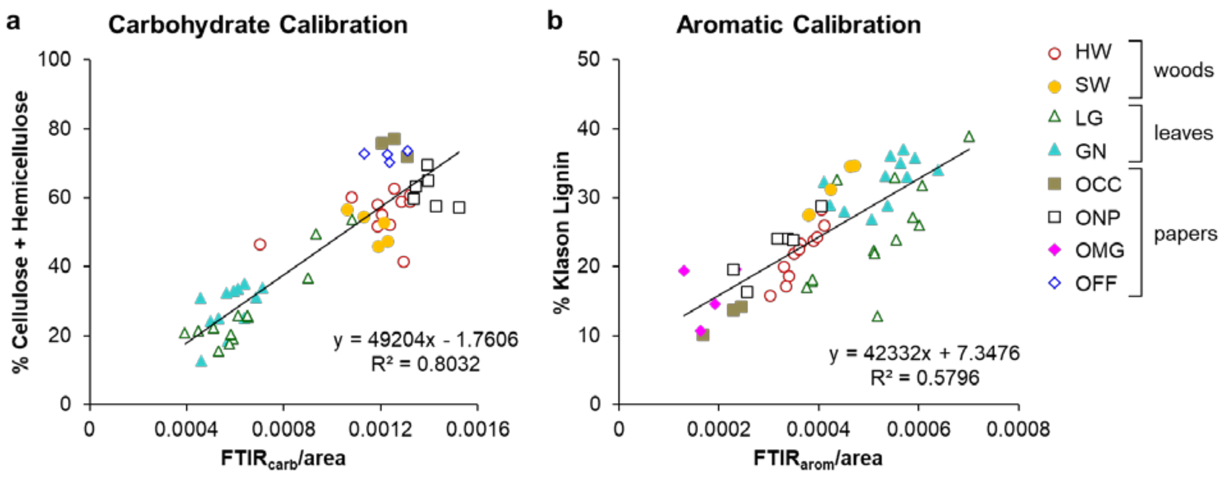

Supplementary Fig. 2 Calibration of peaks used to derive carbohydrate and aromatic contents. Fourier transform infrared spectroscopy (FTIR) measurements (x-axes) represent area-normalized baseline-corrected peak heights (see Methods; Supplementary Fig. 1, Supplementary Table 1), and the wet chemistry measurements (y-axes) are weight percentages. Calibrations are shown for (a) the FTIR carb peak with % cellulose + hemicellulose measured by acid hydrolysis and HPLC (n = 54), and (b) the FTIR arom15 + arom16 peaks with % Klason lignin (n = 54). Details on the standard set and wet chemistry methods are given in De la Cruz *et al.*[1]. Abbreviations: HW=hardwood, SW=softwood, LG=leaves and grasses, GN=gymnosperm needles, OCC=old corrugated cardboard, ONP=old newsprint, OMG=old magazines, OFF=office paper.

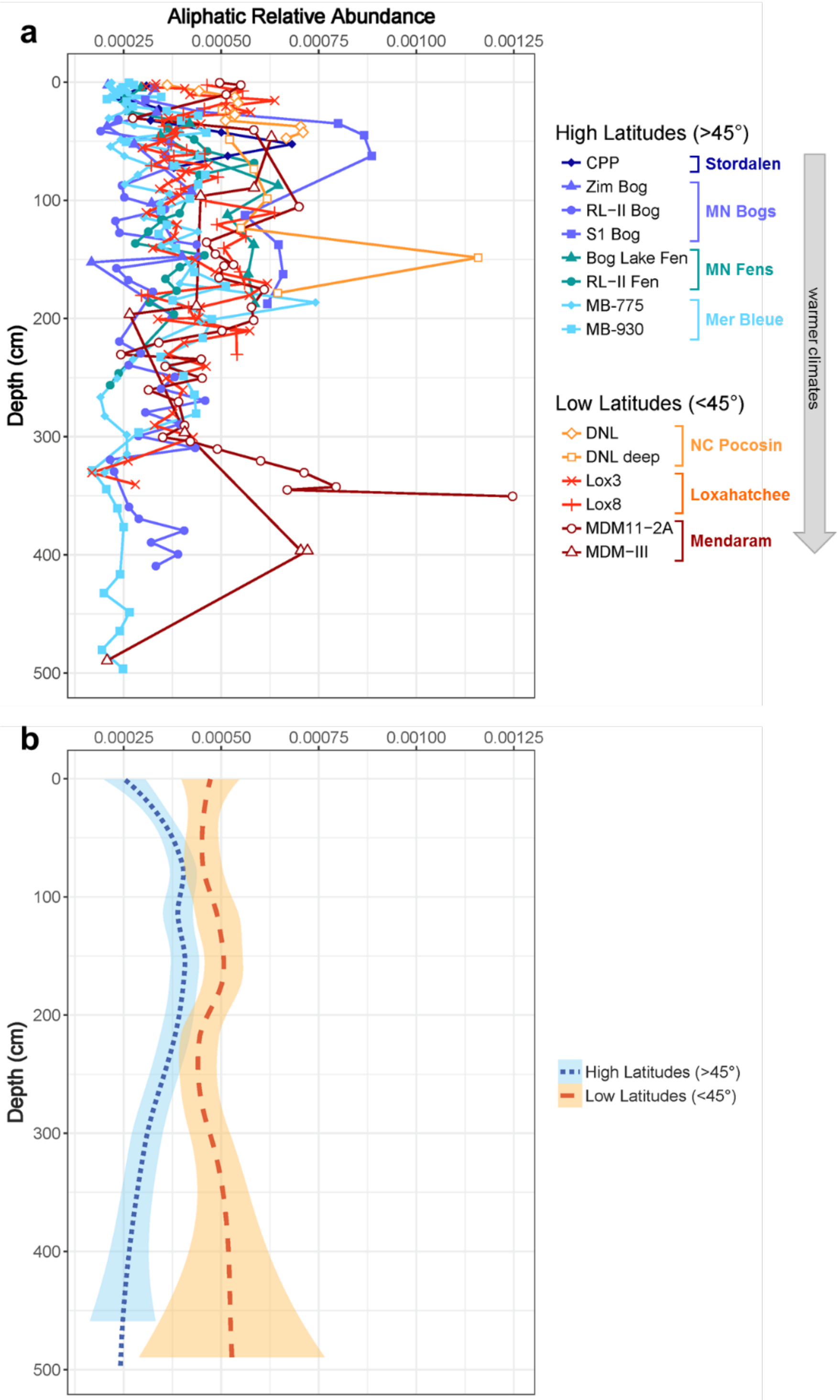

Supplementary Fig. 3 Depth profiles of aliphatic relative abundance in peat from across the latitudinal transect. These relative abundances were calculated as the baseline-corrected peak height at ~2920 cm^{-1} (aliph29 in Supplementary Fig. 1) normalized to the integrated area of the spectrum. (a) Individual depth profiles of aliphatic relative abundance. (b) General trends for high- and low-latitude peatlands illustrated with locally-weighted polynomial regression (LOESS) smooth curves and shaded 95% confidence intervals (LOESS parameters: degree=2, α=0.75) for the profiles shown in (a).

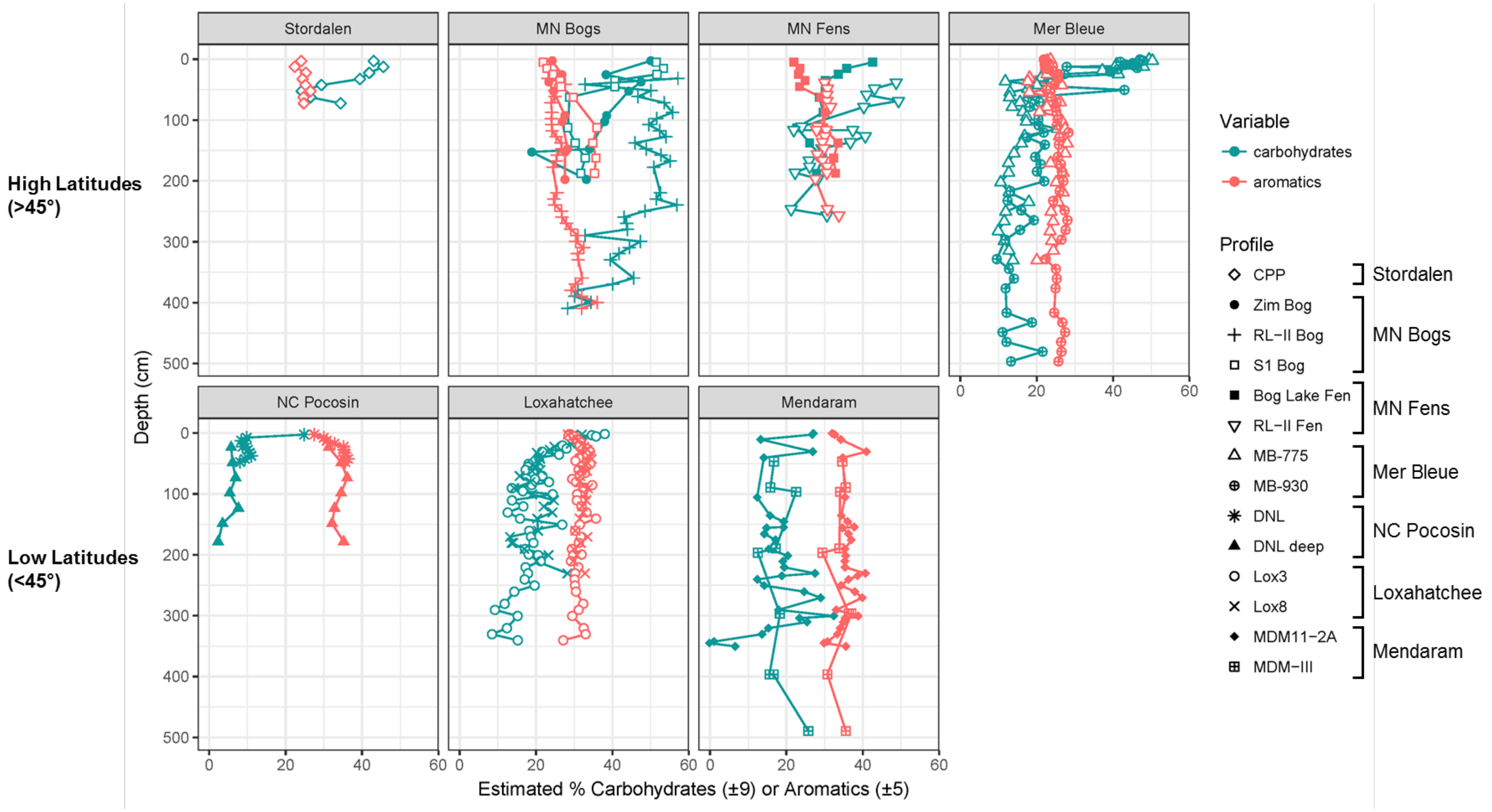

Supplementary Fig. 4 Variations in peat chemistry depth profiles across the latitudinal transect. Estimated weight percentages of carbohydrates and aromatics were determined based on Fourier transform infrared spectroscopy (FTIR) peak heights calibrated to wet chemistry measurements (see Methods), and are shown separated by site category with carbohydrates and aromatics on the same graphs. The same depth profiles are also shown separated by measured variable (carbohydrates or aromatics) in Fig. 2ab. Errors listed in the x-axis are the standard errors of the y estimates for the calibrations shown in Supplementary Fig. 2.

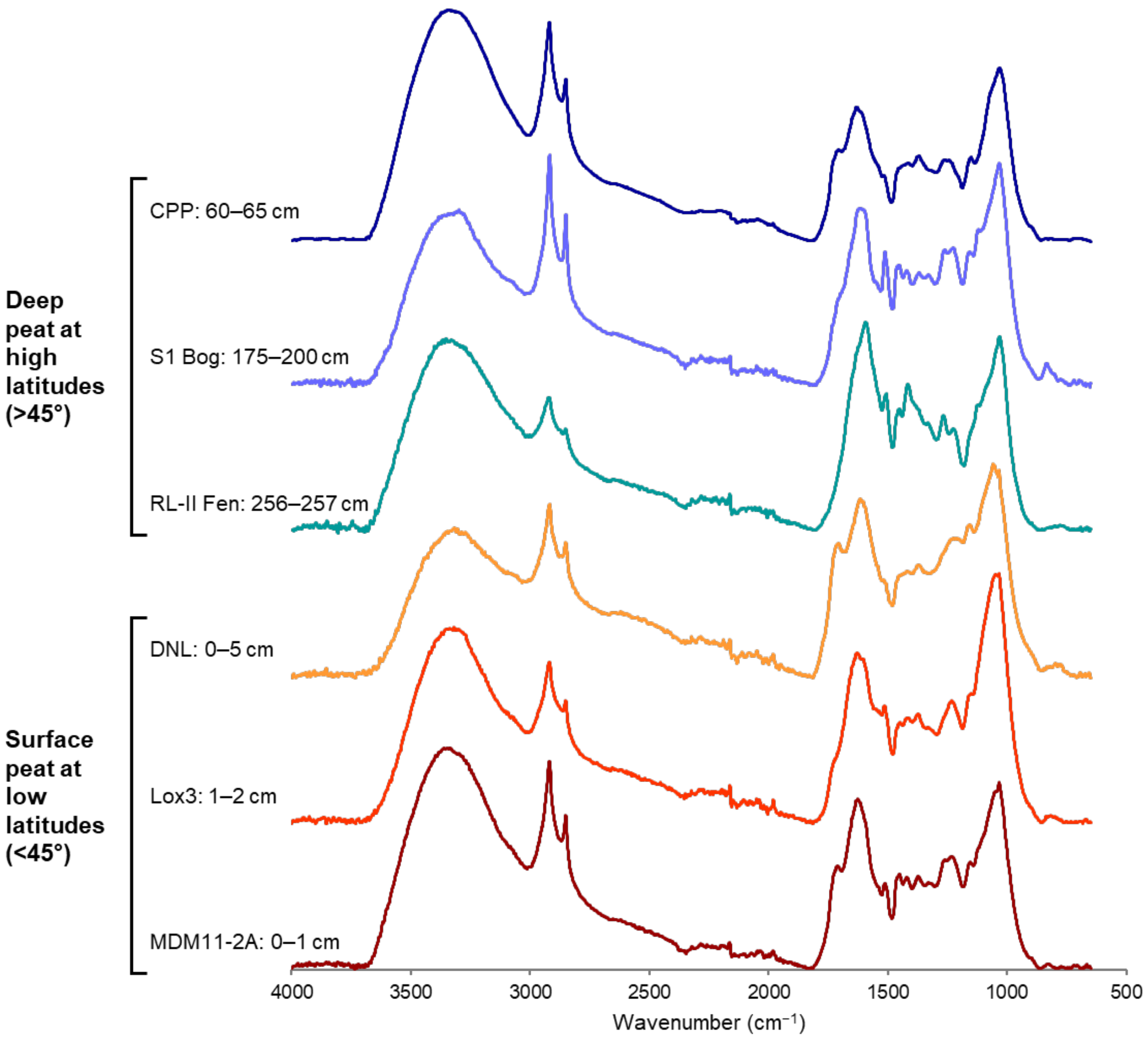

Supplementary Fig. 5 Fourier transform infrared spectra of representative samples of deep peat at high latitudes and surface peat at low latitudes. All spectra have been normalized to constant area, and have estimated carbohydrate and aromatic contents ranging from 25–38% and 25–35%, respectively.

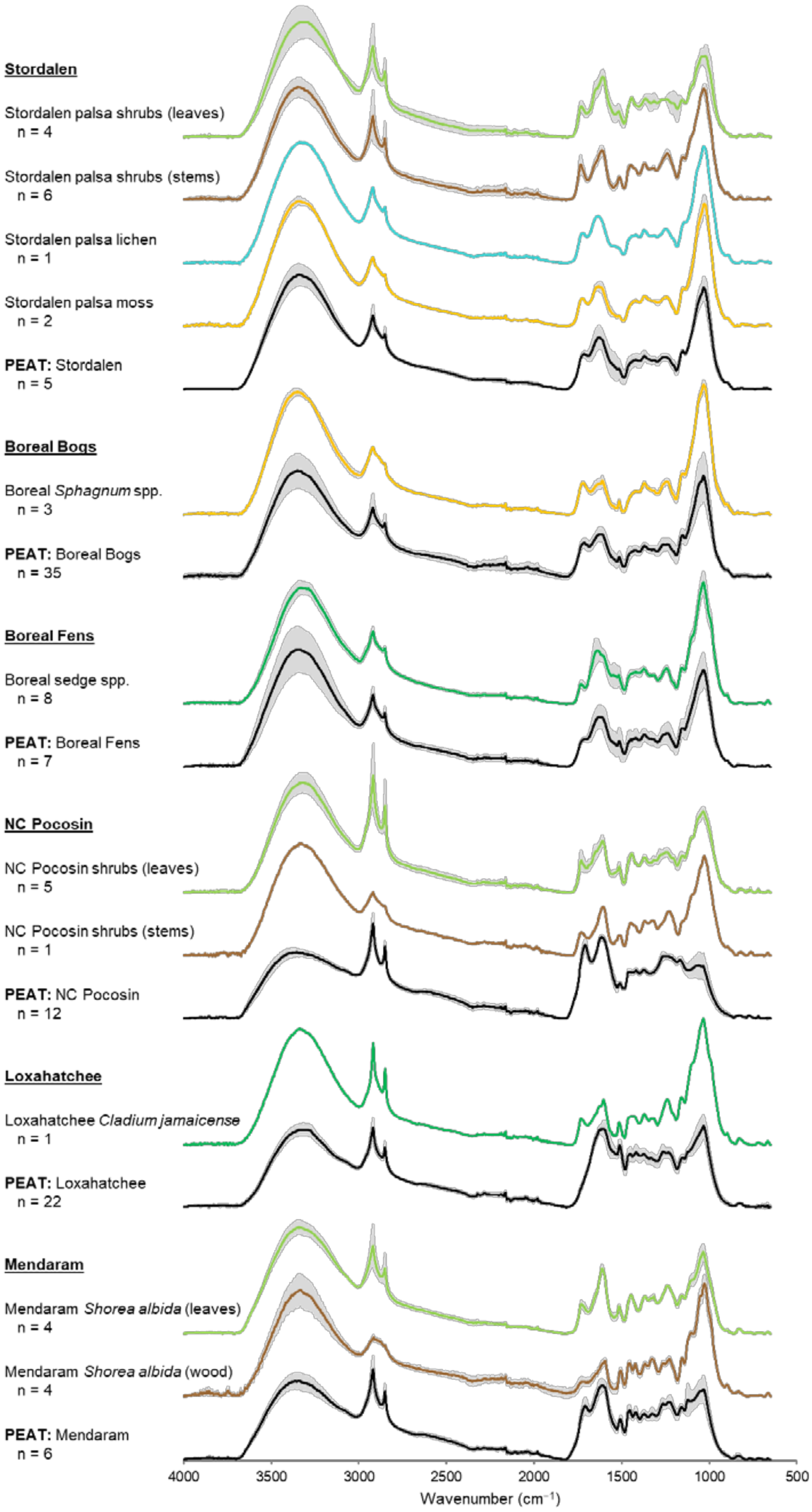

Supplementary Fig. 6 Fourier transform infrared spectra of plants and surface peat. Estimated carbohydrate and aromatic contents derived from these spectra are summarized in Fig. 5. Spectra are grouped by peat source region (Supplementary Tables 2 and 3), with plants further separated by functional type and plant part, and subgroups with *n* > 1 are shown as averages ± standard deviations (one SD). All spectra have been normalized to constant area.

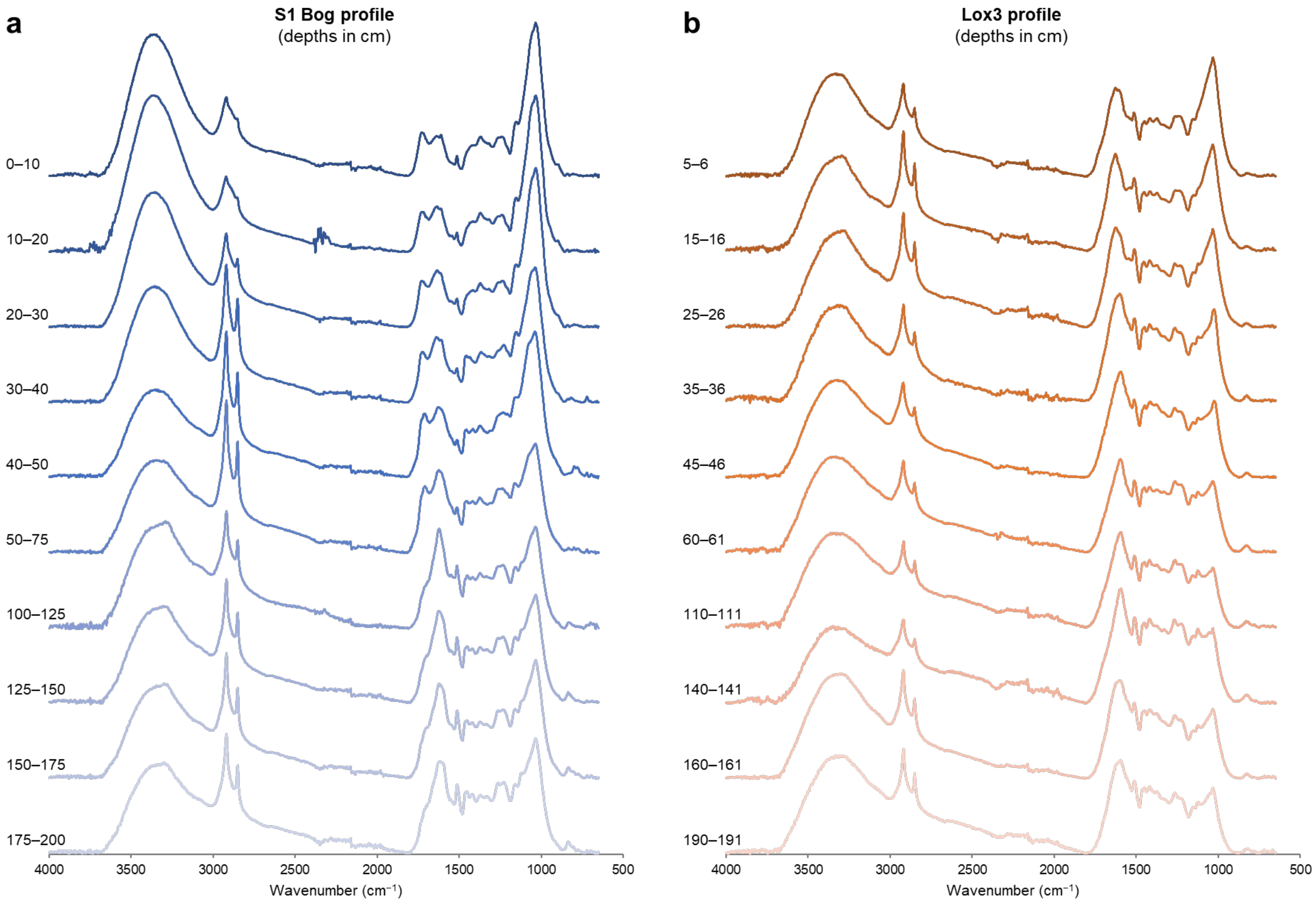

Supplementary Fig. 7 Comparison of Fourier transform infrared spectra for several peat depths from high and low latitudes. Depths (cm) are selected to be similar between cores, which include (a) S1 Bog (high latitude) and (b) Lox3 (low latitude). All spectra have been normalized to constant area.

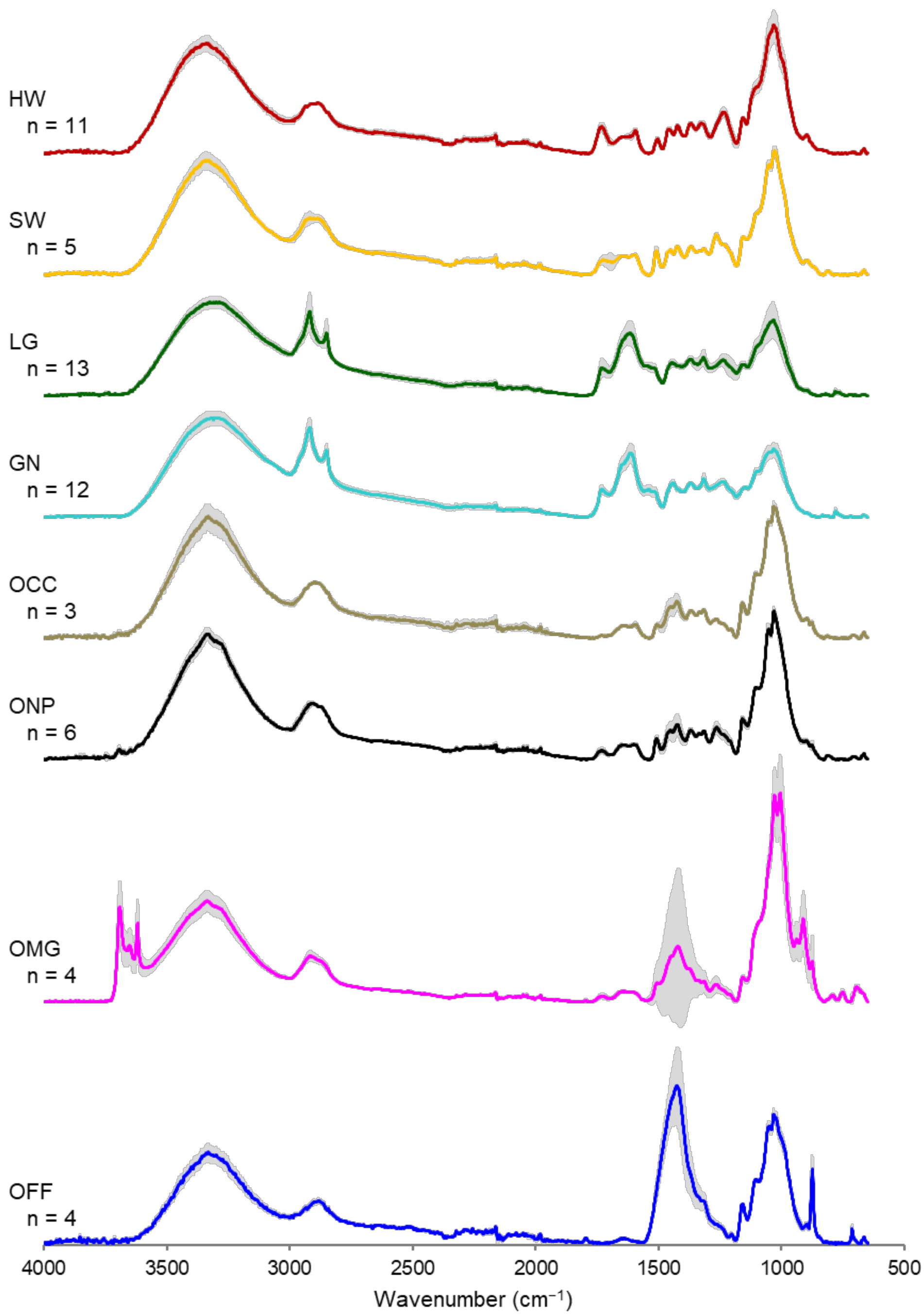

Supplementary Fig. 8 Average Fourier transform infrared spectra of calibration standards. Spectra are grouped by category (same color scheme as Supplementary Fig. 2), with shaded areas representing standard deviations (one SD). Abbreviations: HW=hardwood, SW=softwood, LG=leaves and grasses, GN=gymnosperm needles, OCC=old corrugated cardboard, ONP=old newsprint, OMG=old magazines, OFF=office paper. All spectra have been normalized to constant area. Despite the unusual features in the OMG and OFF samples, their estimated aromatic and carbohydrate contents (respectively) still aligned with the other samples along the calibration curves (Supplementary Fig. 2).

Supplementary Tables

Supplementary Table 1 Wavenumbers of Fourier transform infrared spectroscopy peaks and baseline endpoints measured in this study. These peaks and baseline endpoints (w_1 = lower wavenumber; w_2 = upper wavenumber) are shown graphically in Supplementary Fig. 1. Values in parentheses represent experimentally-determined averages ± standard deviations (one SD). For the carb, arom15, and arom16 peaks, these were calculated as weighted averages ± SD, weighted equally between the peat samples and calibration standards (omitting standards not used to calibrate each peak; see Methods). Because the aliph29 peak was not calibrated, its averages ± SD include only the peat samples.

Peak	Structural Assignment	Peak wavenumbers (cm^{-1})	w_1 (cm^{-1})	w_2 (cm^{-1})
carb	carbohydrates	~1030 (1032 ± 3)	890–920 (915 ± 6)	1150–1210 (1184 ± 4)
arom15	aromatic C=C	~1510 (1510 ± 4)	1470–1500 (1484 ± 3)	1515–1540 (1531 ± 4)
arom16	aromatics, or deprotonated COO^-	~1615 (1615 ± 15)	[w_2 of arom15]–1590 (1536 ± 12)	1760–1850* (1788 ± 15)
aliph29	lipids, waxes, and other aliphatics	~2920† (2919 ± 1)	2750 (constant)	2950–3050 (3005 ± 10)

* Lowest wavenumber in the range 1760–1850 cm^{-1} with absorbance ≤ [local minimum in this range] + [0.01 × maximum absorbance at wavenumbers >1800 cm^{-1}]. This adjustment, which sets w_2 of arom16 equal to the lowest wavenumber with an absorbance close to the minimum, is done because the local minimum is often at too high a wavenumber, such that the baseline actually passes through part of the spectrum close to this endpoint.

† The wavenumber of aliph29 is actually assigned to the largest peak between w_1 and w_2. In all the peat samples measured in this study, as with most natural organic matter, this peak occurred at ~2920 cm^{-1}.

Supplementary Table 2 Climate characteristics and metadata for surface peat. These samples (from the top 50 cm of cores) are analyzed based on climate characteristics in Fig. 3 and compared with plants in Fig. 5. All temperatures are averages of monthly data from the indicated weather station and date range.

Region	Core	Depth range (cm)	n	Latitude (°N)	Mean annual temperature (°C)	Temperature Source
Stordalen	CPP	1–45	5	68.3531	−0.5	Abisko Scientific Research Station, 1950–2010 (Annika Kristoffersson, personal communication)
Minnesota: MN Bogs	Zim Bog	0–50	3	47.1791	3.4	Hibbing Chisholm Airport, 1971–2000[2]
Minnesota: MN Bogs	RL-II Bog	31–42	2	48.2547	3.3	Waskish 4 NE, 1971–2000[2]
Minnesota: MN Bogs	S1 Bog	0–50	5	47.5063	4.2	Grand Rapids Forest Lab, 1971–2000[2]
Minnesota: MN Fens	Bog Lake Fen	0–50	5	47.5051	4.2	Grand Rapids Forest Lab, 1971–2000[2]
Minnesota: MN Fens	RL-II Fen	38–49	2	48.2897	3.3	Waskish 4 NE, 1971–2000[2]
Mer Bleue	MB-775	0–49	11	45.4088	6.0	Ottawa Macdonald-Cartier International Airport, 1971–2000[3]
Mer Bleue	MB-930	0–43	14	45.4110	6.0	Ottawa Macdonald-Cartier International Airport, 1971–2000[3]
NC Pocosin	DNL	0–50	10	35.6905	16.7	Plymouth 5 E, 1971–2000[2]
NC Pocosin	DNL deep	22–50	2	35.6904	16.7	Plymouth 5 E, 1971–2000[2]
Loxahatchee	Lox3	1–46	12	26.5967	24.1	West Palm Beach International Airport, 1971–2000[2]
Loxahatchee	Lox8	0–50	10	26.5200	24.1	West Palm Beach International Airport, 1971–2000[2]
Mendaram	MDM11-2A	0–41	5	4.3727	27.1	Seria and Kuala Belait stations, 1947–2004[4]
Mendaram	MDM-III	43–50	1	4.3702	27.1	Seria and Kuala Belait stations, 1947–2004[4]

Supplementary Table 3 Metadata for plants used for comparison with peat. These comparisons of plant and peat chemistry are shown in Fig. 4cd and Fig. 5.

Species	Plant Part	Sampling Date	n	Site of Plant Collection	Sites Used for Peat Comparison
Andromeda polifolia	leaf	June 2015	1	Stordalen (palsa)	Stordalen
Andromeda polifolia	stem	June 2015	1	Stordalen (palsa)	Stordalen
Rubus chamaemorus	leaf	June 2015	1	Stordalen (palsa)	Stordalen
Rubus chamaemorus	leaf	July 2015	1	Stordalen (palsa)	Stordalen
Rubus chamaemorus	stem	June 2015	2	Stordalen (palsa)	Stordalen
Empetrum nigrum	leaf	July 2015	1	Stordalen (palsa)	Stordalen
Empetrum nigrum	stem	July 2015	1	Stordalen (palsa)	Stordalen
Betula nana	stem	June 2015	1	Stordalen (palsa)	Stordalen
Vaccinium vitis-idaea	stem	June 2015	1	Stordalen (palsa)	Stordalen
Lichen (unidentified)	whole plant	June 2015	1	Stordalen (palsa)	Stordalen
Moss (unidentified)	whole plant	June 2015	1	Stordalen (palsa)	Stordalen
Sphagnum fuscum	whole plant	June 2015	1	Stordalen (palsa)	Stordalen
Sphagnum (unidentified)	whole plant	July 2012	1	S1 Bog	Boreal Bogs
Sphagnum angustifolium	whole plant	May 2016	1	S1 Bog	Boreal Bogs
Sphagnum magellanicum	whole plant	May 2016	1	S1 Bog	Boreal Bogs
Eriophorum vaginatum	stem + leaf	May 2016	1	S1 Bog	Boreal Fens
Eriophorum viridicarinatum	stem + leaf	May 2016	1	S1 Bog	Boreal Fens
Carex trisperma	stem + leaf	May 2016	1	S1 Bog	Boreal Fens
Carex rotundata	stem + leaf	June 2015	1	Stordalen (fen)	Boreal Fens
Eriophorum angustifolium	stem + leaf	June 2015	2	Stordalen (fen)	Boreal Fens
Eriophorum angustifolium	stem + leaf	July 2015	2	Stordalen (fen)	Boreal Fens
Pinus serotina	leaf	May 2015	1	NC Pocosin	NC Pocosin
Ilex glabra	leaf	May 2015	1	NC Pocosin	NC Pocosin
Persea palustris	leaf	May 2015	1	NC Pocosin	NC Pocosin
Gordonia lasianthus	stem	May 2015	1	NC Pocosin	NC Pocosin
Lyonia lucida	leaf	May 2015	1	NC Pocosin	NC Pocosin
Vaccinium formosum	leaf	May 2015	1	NC Pocosin	NC Pocosin
Cladium mariscus ssp. *jamaicense*	stem + leaf	October 2015	1	Loxahatchee	Loxahatchee
Shorea albida	leaf (intact litter)	Aug. 2016	4	Badas, Brunei	Mendaram
Shorea albida	wood	Feb. 2012 – Aug. 2015 (see Methods)	4	Mendaram	Mendaram

Supplementary References

1. De la Cruz, F. B., Osborne, J. & Barlaz, M. A. Determination of sources of organic matter in solid waste by analysis of phenolic copper oxide oxidation products of lignin. *J. Environ. Eng.* **142,** 04015076 (2016).
2. The NOAA National Environmental Satellite, Data, and Information Service (NESDIS). National Climatic Data Center: U.S. Climate Normals. *National Centers for Environmental Information (NCEI)* Available at: https://www.ncdc.noaa.gov/cgi-bin/climatenormals/climatenormals.pl. (Accessed: 23rd May 2017)
3. Environment and Climate Change Canada. Canadian Climate Normals - Climate - Environment and Climate Change Canada. *Government of Canada* (2011). Available at: http://climate.weather.gc.ca/climate_normals/. (Accessed: 21st November 2017)
4. Dommain, R. *et al.* Forest dynamics and tip-up pools drive pulses of high carbon accumulation rates in a tropical peat dome in Borneo (Southeast Asia). *J. Geophys. Res. Biogeosciences* **120,** 617–640 (2015).
