# Supplementary material for: Tropical peatland carbon storage linked to global latitudinal trends in peat recalcitrance
Source: Nat Commun. 2018 Sep 7;9:3640. doi: 10.1038/s41467-018-06050-2 (PMC6128871; doi:10.1038/s41467-018-06050-2)
Supplement: Supplementary file 3 — Description of Additional Supplementary Files [file 41467_2018_6050_MOESM3_ESM.pdf]

Description of Additional Supplementary Files

File Name: Supplementary Data 1
Description: Complete data from the calibration standards. The first sheet, Sample Info, includes metadata and wet chemistry results for % cellulose + hemicellulose and % Klason lignin (by weight; ref 22). The second sheet, Raw Spectra (absorbance), shows the full Fourier transform infrared (FTIR) spectra. The remaining sheets show the output from the custom R script used to process the FTIR data, with the area-normalized THE FLORIDA STATE UNIVERSITY Dept. of Earth, Ocean and Atmospheric Science • Dept. of Chemistry and Biochemistry 117 N. Woodward Avenue, Tallahassee, Florida 32306-4320 and baseline-corrected peak heights (calibrated with the wet chemistry in Supp. Fig. 2) in the sheet Norm.Corr.Peaks.

File Name: Supplementary Data 2
Description: Complete data from the peat samples. The first sheet, Sample Info, includes metadata and estimated % carbohydrates and % aromatics (by weight; based on the calibrations in Supp. Fig. 2). The second sheet, Raw Spectra (absorbance), shows the full Fourier transform infrared (FTIR) spectra. The remaining sheets show the output from the custom R script used to process the FTIR data, with the area-normalized and baseline-corrected peak heights (used to estimate % carbohydrates and % aromatics) in the sheet Norm.Corr.Peaks.

File Name: Supplementary Data 3
Description: Complete data from the plant samples. The first sheet, Sample Info, includes metadata and estimated % carbohydrates and % aromatics (by weight; based on the calibrations in Supp. Fig. 2). The second sheet, Raw Spectra (absorbance), shows the full Fourier transform infrared (FTIR) spectra. The remaining sheets show the output from the custom R script used to process the FTIR data, with the area-normalized and baseline-corrected peak heights (used to estimate % carbohydrates and % aromatics) in the sheet Norm.Corr.Peaks.

File Name: Supplementary Data 4
Description: Radiocarbon results. Includes metadata, results used to produce Fig. 6, and original data sources.
